# Prevalence and magnitude of muscle loss in the pediatric intensive care unit assessed by ultrasound: a systematic review and meta-analysis

**DOI:** 10.1016/j.jped.2026.101599

**Published:** 2026-09-06

**Authors:** Marcelo de Araújo Nazaré, Olga Naila Ayo Menezes Liberato Franz de Carvalho, Daniel Dominguez Ferraz, Bruno Prata Martinez

**Affiliations:** a Universidade Federal da Bahia, Programa de Pós-Graduação em Medicina e Saúde, Salvador, BA, Brazil; b Hospital Universitário Professor Edgard Santos(HUPES)/HUBrasil, Salvador, BA, Brazil; c Universidade Federal da Bahia, Departmento de Fisioterapia, Salvador, BA, Brazil; d Universidade Federal da Bahia, Programa de Pós-Graduação em Ciências da Reabilitação, Salvador, BA, Brazil; e Universidade do Estado da Bahia, Departamento de Ciências da Vida, Salvador, BA, Brazil

**Keywords:** Pediatrics, Intensive care unit, Ultrasonography, Muscle atrophy, Meta-analysis

## Abstract

**Objective:**

To systematically review and quantify the variation in quadriceps femoris muscle thickness in children admitted to pediatric intensive care units (PICUs) and to identify factors associated with this loss.

**Methods:**

Searches were performed in Cochrane Library, Embase, PubMed, Scopus, and Web of Science databases for studies published without date restrictions. Observational studies assessing quadriceps thickness via ultrasonography at a minimum of two time points during PICU stay were included. The study protocol was registered in PROSPERO (CRD42024628869). Following PRISMA guidelines, data were extracted regarding study characteristics, muscle thickness variations, and associated clinical/demographic factors. Quantitative synthesis utilized random-effects models for percentage change and meta-analysis of proportions for prevalence.

**Results:**

Nine studies (n = 536 patients) were included. The mean reduction in muscle thickness at 7 days was −7.89% (95% CI, −11.36% to −4.41%; p < 0.001). The maximum observed loss during admission was −10.77% (95% CI, −12.87% to −8.67%; p < 0.001). The pooled prevalence of clinically significant atrophy (> 10%) was 49.4% (95% CI, 44.0% to 54.7%). Key factors associated with greater loss included the use of neuromuscular blocking agents, older age (older children vs. infants), and cumulative protein and caloric deficits.

**Conclusions:**

Muscle atrophy in critically ill children is an early, severe, and multifactorial phenomenon, affecting nearly half of the patients. Bedside ultrasonography monitoring is essential to guide early interventions and mitigate long-term functional impairment.

## Introduction

Continuous advances in pediatric critical care have resulted in a significant increase in survival rates for critically ill children over the past few decades [1]. However, this therapeutic success is accompanied by growing concern regarding long-term morbidity, frequently described within the framework of Post-Intensive Care Syndrome-pediatrics (PICS-p) [2]. Among the most impactful components of this syndrome is Intensive Care Unit-acquired weakness (ICU-AW), which is directly associated with the accelerated loss of skeletal muscle mass during the acute phase of critical illness [3,4].

In critically ill children, muscle atrophy occurs early and is severe, triggered by a metabolic cascade involving prolonged immobilization, systemic inflammatory response, oxidative stress, and, often, insufficient nutritional support to meet high catabolic demands [5,6]. Key studies in this field indicate that muscle mass reduction, particularly in the quadriceps femoris muscle, can be detected within the first days of hospitalization [7]. This early loss has been associated with unfavorable clinical outcomes, including prolonged mechanical ventilation (MV) and impaired functional status after hospital discharge [8].

In this context, bedside muscle monitoring has become an essential tool for the multidisciplinary team. Although methods such as muscle biopsy and computed tomography offer high diagnostic accuracy, logistical limitations and risks inherent to the pediatric population make muscle ultrasonography the preferred technique. Ultrasound is a non-invasive, reproducible, and radiation-free method that allows for serial assessment of muscle thickness and quality in real-time, without the need for patient transport [9,10].

Despite growing interest in the scientific literature, considerable heterogeneity is observed in reported results. While some studies demonstrate substantial muscle loss correlated with cumulative protein deficits [6], others found no significant reductions in muscle thickness, suggesting that changes in muscle quality (echogenicity) may precede or be more sensitive than volume loss in certain pediatric populations [11]. Additionally, the influence of associated factors on the severity of atrophy still lacks a robust quantitative synthesis.

To date, the exact magnitude of quadriceps femoris muscle loss in pediatrics and the consistency of this evidence across different pediatric intensive care settings have not been fully consolidated through updated review and meta-analysis methods. This knowledge gap hinders the implementation of targeted early intervention protocols and nutritional support. Therefore, this systematic review with meta-analysis aimed to summarize and quantify the variation in quadriceps femoris muscle thickness, defined as the relative percentage change from baseline admission measurements across serial ultrasound evaluations, in children admitted to pediatric intensive care units (PICUs). Additionally, we sought to identify and categorize clinical, pharmacological, nutritional, and demographic factors associated with the trajectory of muscle loss during critical illness.

## Methods

### Study design and registration

This systematic review and meta-analysis was conducted in accordance with the Preferred Reporting Items for Systematic Reviews and Meta-Analyses (PRISMA) guidelines [12]. The protocol was registered in the PROSPERO database (registration number: CRD42024628869) to ensure transparency and avoid duplication of efforts.

### Eligibility criteria

Study selection followed the PEO (Population, Exposure, and Outcome) framework: [13]

− Population: Children and adolescents (aged 1 month to 18 years) admitted to pediatric intensive care units (PICUs).
− Exposure: Admission to a critical care environment.
− Outcome: Variation in quadriceps femoris muscle thickness measured by ultrasonography (US) at a minimum of two distinct time points during the PICU stay.

**Inclusion Criteria:** Observational studies (prospective cohorts) providing quantitative data on muscle thickness.

**Exclusion Criteria:** Studies involving exclusively adult populations.

#### Search strategy

The search was conducted without language restrictions. The search was conducted without language restrictions. No date restrictions were imposed either, given that this is a relatively recent field of study, with pioneering studies dating back <10 years. The following databases were consulted: Cochrane Library, Embase, PubMed, Scopus, and Web of Science. The search strategy was constructed using MeSH terms and Boolean operators, as detailed below: ("pediatric ICU" OR "pediatric intensive care unit" OR "pediatric intensive therapy unit" OR "neonatal ICU" OR "neonatal intensive care unit" OR “Critically Ill Children” OR “pediatric patients” OR “PICU”) AND ("ultrasonography" OR "ultrasound" OR "muscle ultrasound") AND ("muscle loss" OR "muscle atrophy" OR "muscle wasting" OR "muscle mass" OR "sarcopenia" OR “muscle” OR “thickness”). The specific search strategy for each database is provided in the supplemental digital content.

### Selection and data extraction

Identified articles were exported to the Rayyan platform for study selection and management [14]. Initially, the tool was used to identify and remove duplicates. Subsequently, title and abstract screening was performed independently by two reviewers, utilizing the blinding feature to prevent selection bias. Disagreements were resolved by a third reviewer. For each article excluded during this phase or during the full-text review, the reasons for exclusion were recorded directly on the platform, following the PRISMA Statement guidelines [12].

Data extraction was performed independently and in a standardized manner by two reviewers, collecting: (a) Study identification; (b) Sample characteristics (n, age, diagnosis); (c) US protocol; (d) Associated factors; (e) Primary outcomes (percentage variation in thickness); and (f) Results of associated factors. Subsequently, the independently extracted data were merged, and any divergent information was resolved by consensus.

### Risk of bias assessment

Risk of bias and methodological quality were assessed using the Newcastle-Ottawa Scale (NOS) for cohort studies. Three domains were evaluated: (1) Selection of cohorts; (2) Comparability (adjustment for confounding variables); and (3) Outcome (assessment of outcome and follow-up duration). Studies with scores ≥ 7 stars were considered high quality, 4–6 stars moderate quality, and < 4 stars low quality. Publication bias was qualitatively assessed through visual inspection of funnel plots.

### Statistical analysis

Statistical analyses were conducted using jamovi software (version 2.6) with the MAJOR module [15]. A random-effects model with the Restricted Maximum Likelihood (REML) estimator was used for all outcomes. For studies that reported dispersion data as median and interquartile range, values were converted to mean and standard deviation following the estimation methods proposed by Wan et al. [16]. The quantitative synthesis encompassed continuous variables regarding the percentage change in muscle thickness (assessed at the ∼7-day time point and at the maximum recorded loss) and categorical variables through a meta-analysis of proportions to determine the prevalence of clinically significant muscle atrophy, defined as a loss greater than 10% from baseline. In cases where prevalence was not directly reported, values were estimated through individual patient data analysis (when provided by the authors) or via Z-score based estimation derived from quantitative muscle loss. Statistical heterogeneity was evaluated using Cochran’s Q test and quantified by the I^2^ index. Publication bias was assessed qualitatively by visual inspection of the funnel plot and quantitatively using Kendall's rank correlation test and Egger's regression test. The level of statistical significance was set at p < 0.05.

## Results

### Study selection

**Figure 1:**
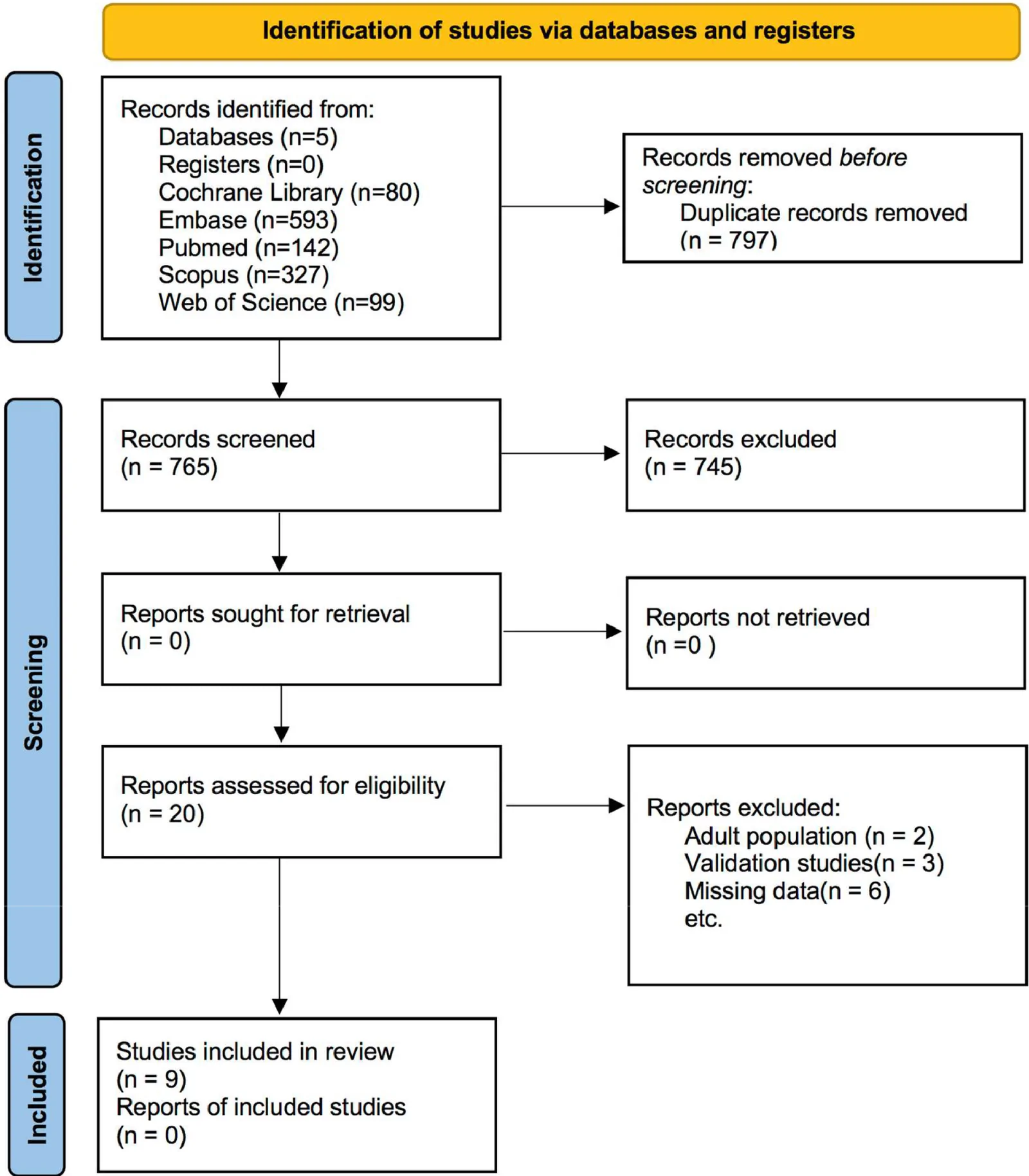
PRISMA flow diagram of the study selection process.

The systematic database search initially identified a total of 1241 records. After the removal of duplicates, 765 articles were screened by title and abstract. Following this phase, 20 articles were selected for full-text review. Ultimately, 9 prospective observational studies met all eligibility criteria and were included in both the qualitative and quantitative synthesis. The detailed selection flow, in accordance with the PRISMA 2020 recommendations, is presented in Figure 1.

### Study and population characteristics

The included studies were published between 2017 and 2024, representing a diverse global population from Brazil (2), the United States (2), France (1), the United Kingdom (1), India (1), Spain (1), and Singapore (1). The combined total sample size comprised 536 pediatric patients.

Ages ranged from full-term neonates to 18-year-old adolescents. Regarding clinical status, seven out of nine studies (77.7%) exclusively included patients undergoing invasive mechanical ventilation. The quadriceps femoris muscle was the primary assessment target in all studies, utilizing high-frequency linear transducers (7.5 to 12 MHz). The characteristics of each study are summarized in Table 1.

**Table 1:** Characteristics of included studies. MV: mechanical ventilation; NMBA: Neuromuscular blocking agents; PICU: pediatric intensive care unit; QF: Quadriceps femoris; TBI: traumati.

Study	Country	n	Participant Characteristics	Age	Assessment Timepoints	Max Muscle Loss	Atrophy Prevalence (> 10%)	Conclusions
Valla et al. (2017)	France	35	Critically ill on MV > 48 h; medical and surgical	2.5 years (Median)	Admission and 5th day	13.30%	59.5%	Early and frequent muscle loss; ultrasound is a reliable monitoring tool.
Johnson et al. (2018)	USA	34	PICU patients with high TBI rates	5.42 years (Mean)	Admission and weekly	8.62%	53.0%	Early and frequent muscle loss; older age and TBI may increase atrophy severity.
Figueiredo et al. (2021)	Brazil	119	General PICU; medical and surgical	1 year (Median)	Admission and 7th day	16.35%	58.2%	Early and frequent muscle loss; potentially related to insufficient protein intake. Ultrasound is promising for sarcopenia assessment.
Hoffmann et al. (2021)	Brazil	36	Patients on MV with nutritional support	1.2 years (Median)	Admission and daily	8.20%	41.9%	Early and frequent muscle loss; associated with nutritional intake, not confounded by fluid balance; QF ultrasound is reliable.
Ong et al. (2021)	Singapore	73	Respiratory failure on MV	4.94 years (Mean)	Critical period	8.40%	43.5%	Muscle loss is associated with energy inadequacy and impaired post-discharge muscle growth; changes correlated with mobility and quality of life.
Jain et al. (2022)	India	58	Patients with sepsis and septic shock	4.2 years (Mean)	Admission and 7th day	5.70%	Could not be estimated	Echogenicity may be a more sensitive marker than thickness for assessing muscle properties in critically ill children.
Montoro et al. (2023)	Spain	41	Prolonged MV and high exposure to NMBAs	6 months (Median)	Admission, 72 h, and 7th day	12.50%	43.9%	Atrophy strongly associated with NMBA use; bedside ultrasound can identify skeletal muscle atrophy.
Oliveira et al. (2024)	Brazil	97	General PICU; medical and surgical	1.3 years (Median)	Admission, 72 h, 7th day, and 14th day	10.75%	46.2%	Significant prevalence of atrophy at the end of 2 weeks in the PICU, regardless of age and diagnosis.
Tume et al. (2024)	UK	34	Patients on MV; focus on inflammation	2.2 years (Median)	Admission, 72 h, and 7th day	11.29%	44.0%	Muscle loss observed, but no association between 72 h protein/energy intake and QF muscle loss.

MV, Mechanical Ventilation; NMBA, Neuromuscular Blocking Agents; PICU, Pediatric Intensive Care Unit; QF, Quadriceps Femoris; TBI, Traumatic Brain Injury.

### Methodological quality and risk of bias

Assessment using the Newcastle-Ottawa Scale (NOS) revealed that the included studies exhibit moderate methodological quality, with scores ranging from 4 to 6 stars (Table 2 with all studies methodological quality assessment using the Newcastle-Ottawa Scale (NOS) for cohort studies available in the digital supplemental content).

**Table 2:** Methodological quality assessment using the Newcastle-Ottawa scale (NOS) for cohort studies c brain injury.

Study	Selection				Comparability	Outcome			Total
	1	2	3	4	1	1	2	3	
Valla et al., 2017	★	−	★	−	★	★	★	★	6
Johnson et al., 2018	−	−	★	−	★	★	★	★	5
Figueiredo et al., 2021	−	−	★	−	★	★	★	−	4
Hoffmann et al., 2021	−	−	★	−	★	★	★	★	5
Ong et al., 2021	−	−	★	−	★	★	★	★	5
Jain, 2022	−	−	★	−	★	★	★	−	4
Montoro et al., 2023	−	−	★	−	★	★	★	★	5
Oliveira et al., 2024	−	−	★	−	★	★	★	−	4
Tume et al., 2024	−	−	★	−	★	★	★	★	5

**Selection:** 1) Representativeness of the exposed cohort; 2) Selection of the non exposed cohort; 3) Ascertainment of exposure 4) Demonstration that outcome of interest was not present at start of study.

**Comparability:** 1) Comparability of cohorts on the basis of the design or analysis.

**Outcome:** 1) Assessment of outcome; 2) Was follow-up long enough for outcomes to occur; 3) Adequacy of follow up of cohorts.

### Muscle thickness variation outcome

**Figure 2:**
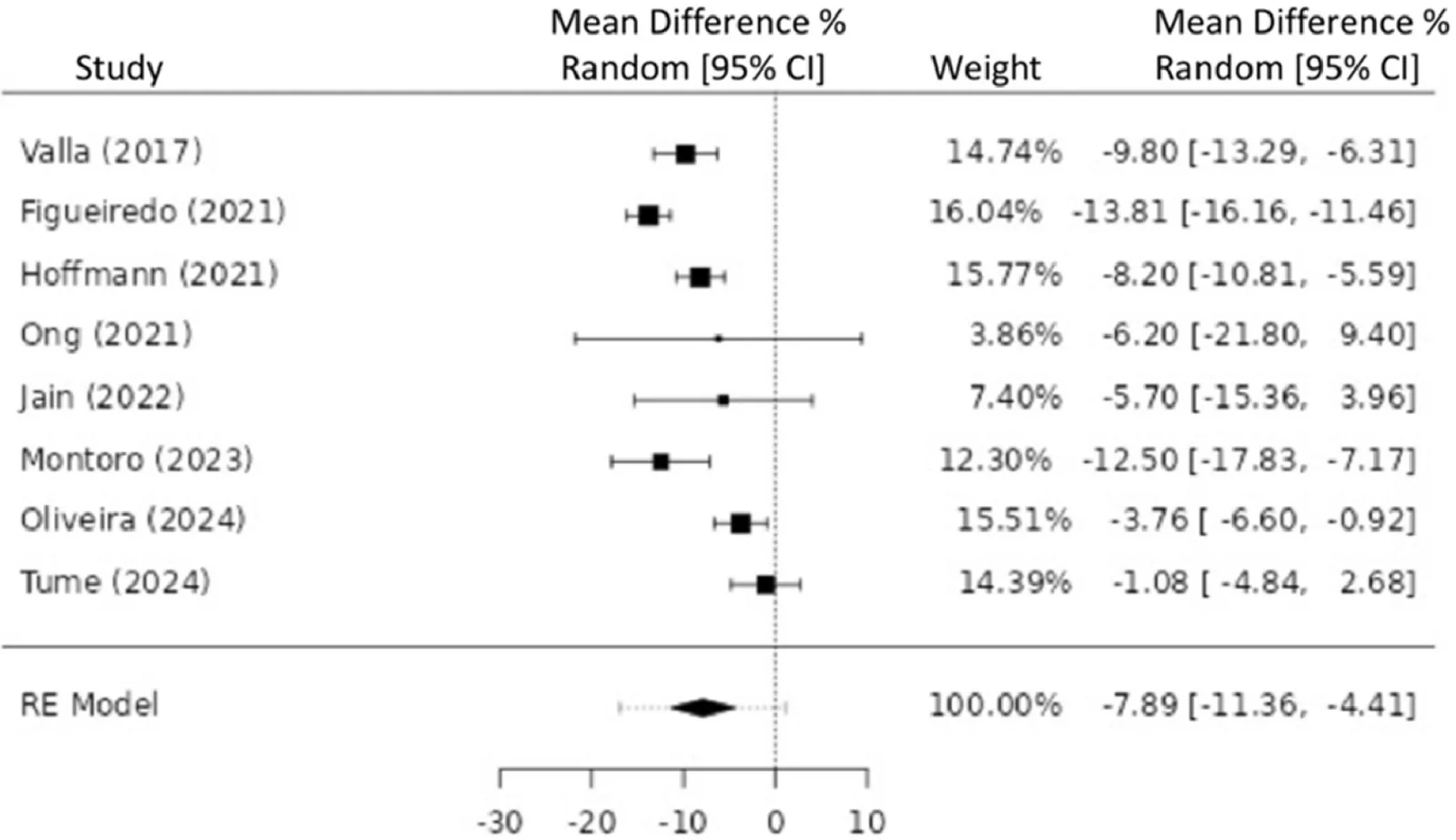
Forest plot of muscle mass loss at 7 days of PICU admission.

**Seven-day Loss:** The random-effects meta-analysis evaluating muscle loss during the first week of admission demonstrated a pooled mean reduction of −7.89% (95% CI: −11.36% to −4.41%; p < 0.001). Statistical heterogeneity was I^2^ = 84,03%, reflecting differences in patient populations and clinical severity across centers. The study by Figueiredo et al. (2021)[6] reported the greatest loss during this period (−13.81%), whereas Tume et al. (2024)[5] and Oliveira et al. (2024)[9] reported more modest reductions (Figure 2).

**Figure 3:**
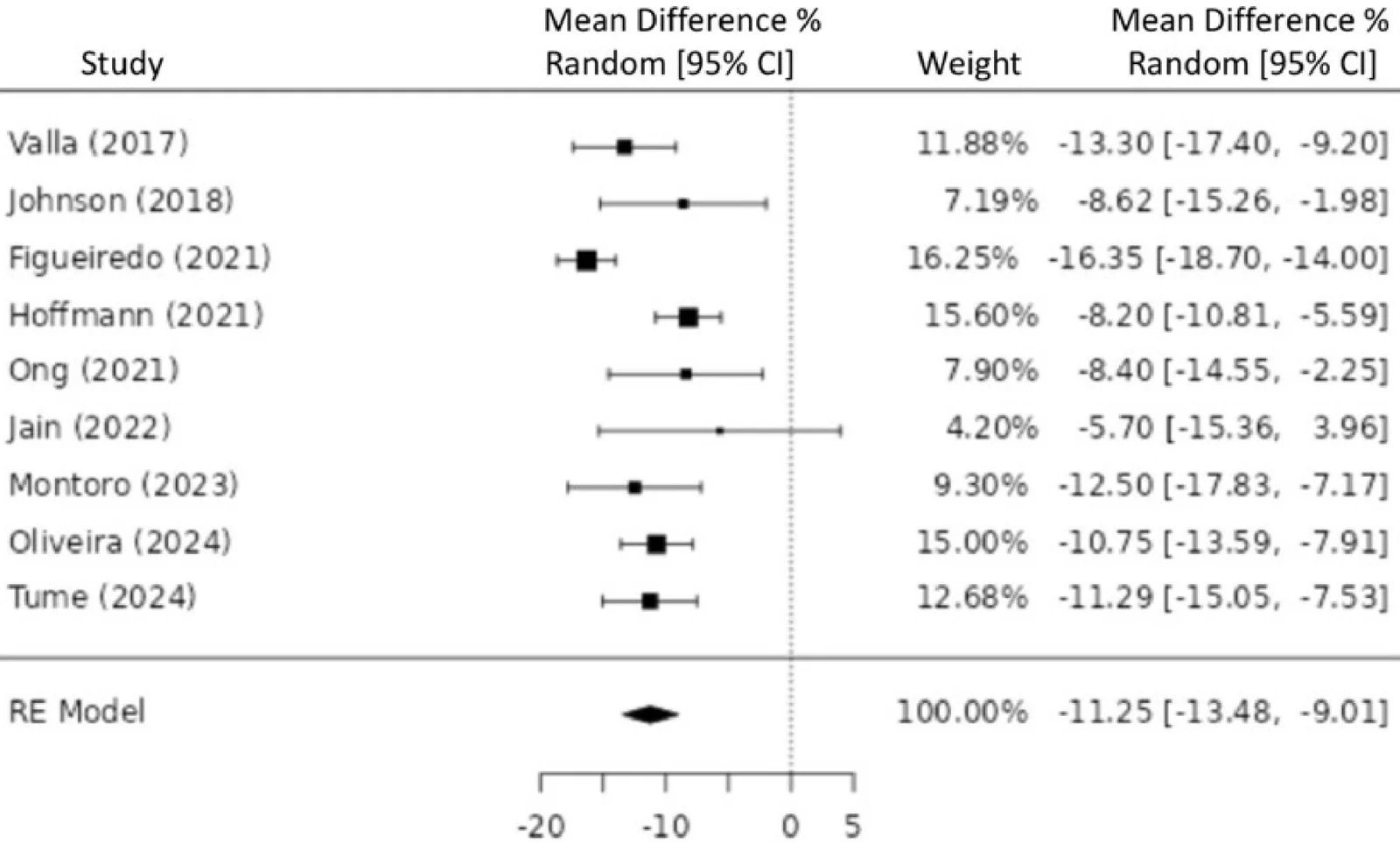
Forest plot of the maximum observed thickness loss at any point during the PICU stay.

**Maximum Observed Thickness Loss:** When analyzing the greatest decline recorded at any point during the PICU stay, the pooled reduction was −10.77% (95% CI: −12.87% to −8.67%; p < 0.001). In this analysis, heterogeneity was I^2^ = 79.43%, indicating greater consistency in the results after scale correction and the inclusion of recent data (Figure 3).

### Prevalence of atrophy outcome

**Figure 4:**
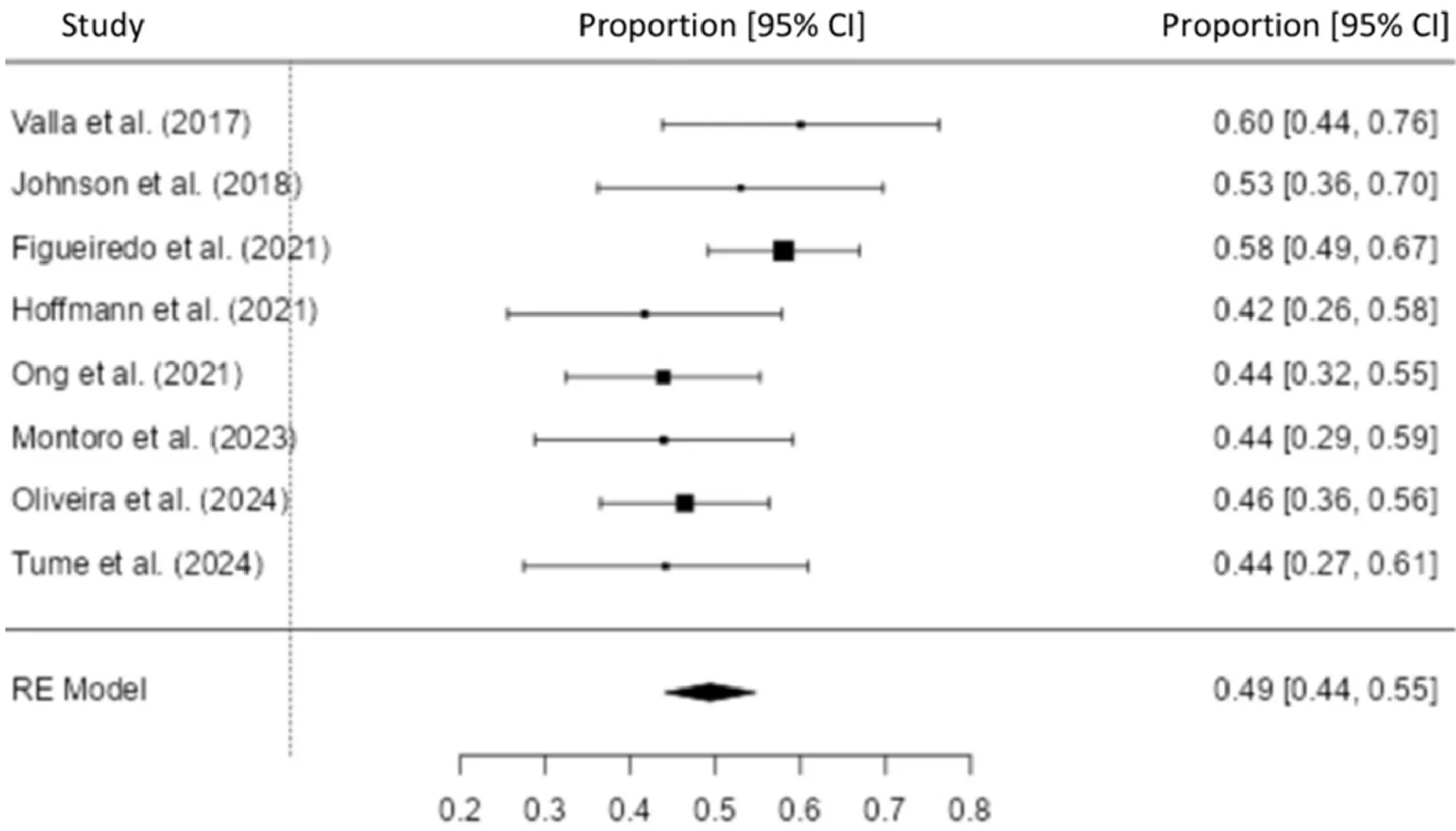
Forest plot of the prevalence of atrophy during PICU admission.

In addition to the mean reduction in muscle thickness, the prevalence of clinically significant muscle atrophy, defined as a loss greater than 10% from baseline, was synthesized using a random-effects meta-analysis (k = 8 studies, as prevalence data were not available in the study by Jain et al., 2022). The estimated pooled prevalence was 49.4% (95% CI: 44.0% to 54.7%; p < 0.001). Unlike the thickness outcome, the prevalence demonstrated low statistical heterogeneity (I^2^ = 24,82%; Q = 8,43; p = 0296), indicating consistency in findings across different centers. Individual studies reported proportions ranging from 42%[10] to 60%[7] (Figure 4).

### Factors associated with muscle atrophy in children admitted to the PICU

The analysis of associated factors revealed that the decline in quadriceps femoris thickness is a multifactorial process, predominantly influenced by nutritional intake, specific pharmacological agents, and demographic/clinical characteristics.

**Nutrition (Protein and Caloric Intake):** While Figueiredo et al. (2021) [6], Hoffmann [10] e Ong [3] confirm that nutritional deficits accelerate the loss of muscle thickness, Tume [5] suggest that this impact may not be immediate within the first 72 h.

**Pharmacology (Neuromuscular Blocking Agents and Corticosteroids):** Montoro et al. (2023) [8] identified that exposure to Neuromuscular Blocking Agents (NMBAs) was the primary predictor of greater muscle loss in multivariate models. Tume et al. (2024) [5] also reported a trend toward greater loss in children exposed to NMBAs, although it did not reach statistical significance. Johnson et al. (2018) [4] and Tume et al. (2024) [5] found no statistically significant associations between the use of intravenous steroids and the magnitude of muscle atrophy in pediatrics.

**Age:** Two studies confirmed that older children experience more severe muscle loss compared to infants [4,8], Specifically, in the study by Montoro et al. (2023), children older than 1 year lost an average of 24.6% in thickness, compared to significantly lower values in infants. Conversely, Oliveira et al. (2024) [9], found that the prevalence of muscle atrophy occurred regardless of age.

**Clinical Diagnosis:** Johnson et al. (2018) [4] highlighted that the presence of Traumatic Brain Injury (TBI) was associated with an additional 18.1% muscle loss. Several studies, including Jain et al., 2022 e Tume et al., 2024[5,11] found no direct correlation between admission severity scores and the intensity of muscle loss.

**Fluid Balance and Edema:** A key methodological point was addressed by Hoffmann et al. (2021) and Jain et al. (2023) [10,11], who evaluated whether a positive fluid balance(aedema) could mask atrophy. Both studies concluded that there was no significant association between cumulative fluid balance and changes in muscle thickness measured by ultrasound.

### Publication bias assessment

**Figure 5:**
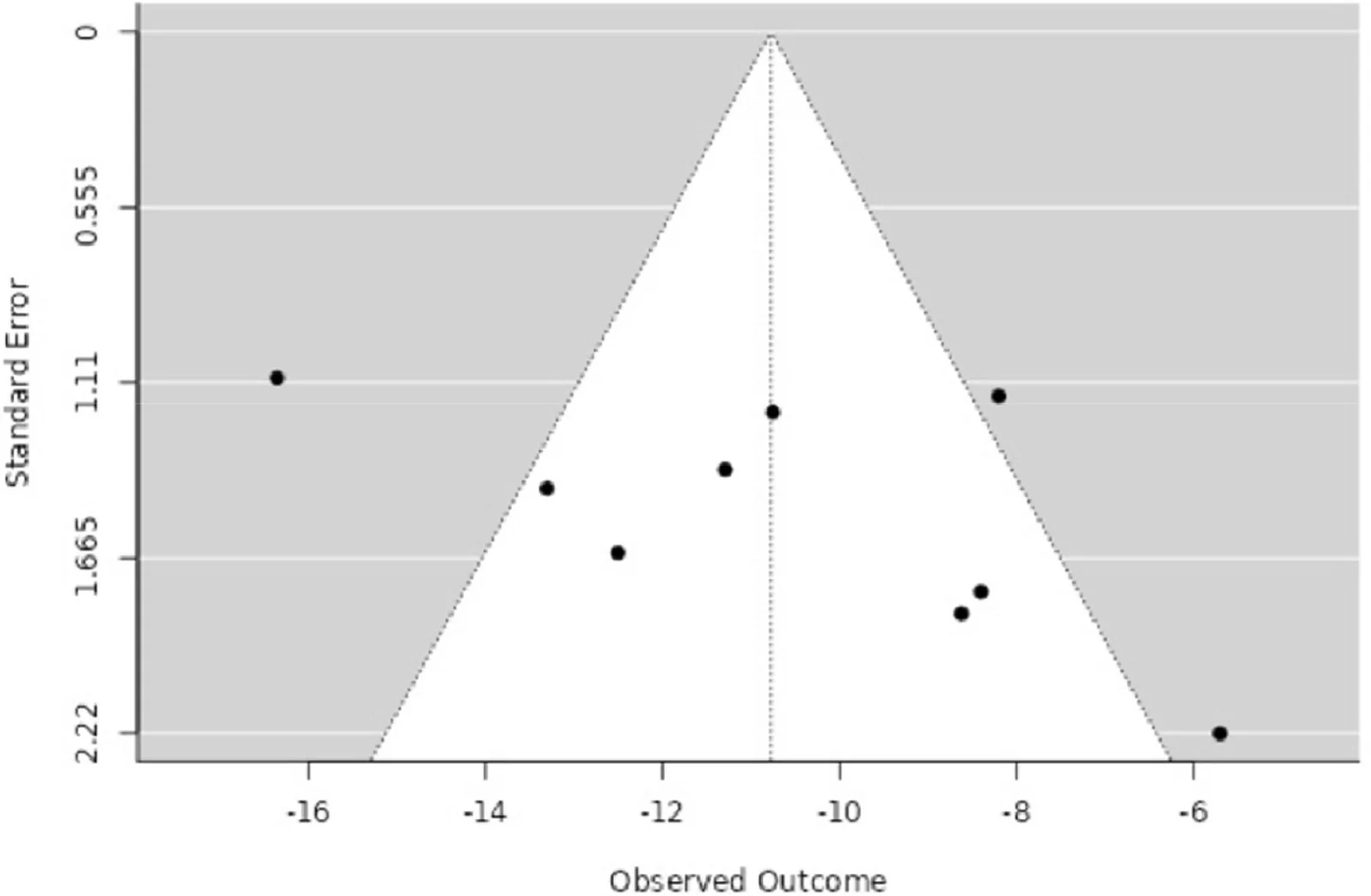
Funnel plot for the assessment of publication bias regarding the muscle loss outcome in children during PICU admission.

Publication bias was assessed using a funnel plot (Figure 5). Although partially limited by the small number of studies, the distribution of the studies exhibited asymmetry, with a higher concentration of publications reporting significant losses. This may reflect the clinical heterogeneity inherent in pediatric samples or a scarcity of studies reporting null results.

## Discussion

This systematic review and meta-analysis synthesizes the current evidence on muscle dynamics in critically ill pediatric patients, revealing that quadriceps femoris atrophy is an early, severe, and multifactorial phenomenon. Our findings demonstrate a pooled mean reduction of 10.77% in muscle thickness during the acute phase, with a striking 49.4% of the studied population experiencing clinically significant losses (> 10%). These data underscore the urgent need to investigate potential associated factors and explore interventions aimed at minimizing these losses and their subsequent consequences.

### Magnitude of atrophy

While the mean thickness loss of 10.77% is numerically lower than the ∼17-20% losses frequently reported in adult critical care [17,18] the functional impact in pediatrics is profound. Children are in a state of continuous growth-related anabolism [19], the disruption of this process by acute critical illness compromises a developing musculoskeletal system [3], potentially impairing future explosive strength and motor coordination [2,20].

Another point of relevance involves the functional recovery trajectory within the spectrum of PICS-p. While rehabilitation for critically ill adults aims to restore a premorbid functional baseline, the challenge in pediatrics is to return the patient to a developmental trajectory that is inherently dynamic and upward [20,21]. The muscle atrophy identified in this meta-analysis may, therefore, manifest as an even more profound functional impact, where delays in achieving fundamental motor milestones (such as sitting, crawling, or walking) generate a cumulative effect on quality of life and social participation [20].

### Prevalence of atrophy

The prevalence of clinically significant muscle atrophy (> 10%) in the pediatric intensive care unit reaches approximately 49.4%, a rate similar to that observed in critically ill adults (40% to 50%) [22]. This similarity counters the historical notion that the innate plasticity and anabolic potential of childhood offer protection against acute catabolism. Our findings emphasize that once the systemic inflammatory response and muscle disuse are established in the PICU, pediatric muscle tissue becomes as vulnerable to degradation as adult tissue, and it degrades rapidly and severely, often within the first week of admission, as noted by recent studies [4,8]. This suggests that the mechanisms of muscle loss operate across all age groups within the critical care setting.

Despite this high incidence, the 49.4% prevalence may be a conservative estimate, as isolated muscle thickness measurements tend to underestimate the actual loss of mass when compared to more advanced imaging methods [23]. Furthermore, there is a crucial difference in the clinical impact thresholds between age groups: while in adults severe negative outcomes [such as prolonged mechanical ventilation and mortality) are associated with muscle losses exceeding 20% [24,25]. In contrast, emerging pediatric evidence suggests that reductions as small as 10% are already predictive of significant long-term functional impairment [26].

## Factors associated with muscle atrophy in children admitted to the PICU

### Pharmacological factors

The use of Neuromuscular Blocking Agents (NMBAs) emerged as a primary predictor of accelerated muscle loss [8]. Chemical paralysis hinders mechanotransduction and aggressively accelerates protein degradation. In contrast to adult cohorts, where corticosteroids are a classic risk factor for myopathy, the evaluated pediatric studies did not show a statistically significant association between intravenous steroids and the magnitude of quadriceps atrophy, suggesting their direct catabolic impact might be masked by systemic inflammation or NMBA use [4,5].

### The role of age and muscle development

Unlike the adult population, where advanced age and preexisting sarcopenia are the primary risk factors, in pediatrics, we observe that older children experience more severe losses. Johnson et al. (2018) [4] demonstrated that each additional year of life is associated with an additional 1.46% decrease in muscle thickness. This may occur because adolescents possess a muscle fiber composition more similar to that of adults (Type II fibers, which are more susceptible to catabolism) compared to infants, whose metabolism is more geared toward accelerated synthesis, conferring a relative biological "protection" against absolute disuse [9].

### Nutrition and anabolic resistance

Nutritional support has been one of the most extensively studied factors, with previous evidence indicating that inadequate delivery accelerates lean mass loss. In general, protein nutrition is described as the traditional defense against catabolism [[27], [28], [29], [30]]. However, our qualitative results reveal a gap: while Figueiredo et al. (2021) [6] and Hoffmann et al. (2021) [10] identified a correlation between protein intake and muscle preservation, Tume et al. (2024) [5] did not observe the same benefit during the first 72 h. This suggests the existence of a phase of anabolic resistance in pediatrics, similar to that observed in adults, where aggressive nutritional delivery in the acute phase may not prevent inflammation-mediated protein degradation [31,32].

### Muscle quality and echogenicity

A critical point lies in the interpretation of muscle thickness measurements. Jain et al. (2022) [11] reported that muscle thickness might not decrease if there is a concurrent increase in echogenicity (muscle quality). In adults, Parry et al. (2015) [33] demonstrated that increased echogenicity reflects fiber necrosis and fat/fluid infiltration. The finding by Jain et al. (2022) [11] of a 16.1% increase in echogenicity indicates that the muscle may maintain its apparent volume due to interstitial edema, even as the contractile architecture is compromised, resulting in a thickness variation of only −5.7%. While Hoffmann et al. (2021) [10] validated that a positive fluid balance does not preclude the detection of actual atrophy, clinicians must remain vigilant to avoid underestimating muscle loss in visibly edematous patients.

### Study limitations

This review is subject to some limitations, most notably the heterogeneity of ultrasound protocols across the included studies, the statistical conversion of medians to means for quantitative synthesis, and the use of prevalence estimates. However, the robustness of the meta-analysis and the consistency observed when benchmarking these findings against adult literature suggest that the data converge toward a solid and reliable finding.

## Conclusion

Based on the findings of this systematic review with meta-analysis, muscle atrophy is an early, severe, and prevalent complication in the pediatric intensive care setting, affecting approximately 49.4% of critically ill patients. Our findings identify the use of NMBAs, older age, and cumulative nutritional deficits as the primary factors associated with this degradation. Therefore, the implementation of bedside muscle ultrasonography should be encouraged as a routine monitoring standard. Such surveillance is essential to guide clinical interventions, integrating nutritional adjustments and early rehabilitation protocols to mitigate the impact of the PICS-p and preserve the developmental growth potential of these children.

## Abreviations and simbols

CI, Confidence Interval; CIPNM, Critical Illness Polyneuromyopathy; CSA, Cross-Sectional Area; ICU, Intensive Care Unit; MV, Mechanical Ventilation; NMBA, Neuromuscular Blocking Agent; NOS, Newcastle-Ottawa Scale; PICS-p, Post-Intensive Care Syndrome-pediatrics; PICU, Pediatric Intensive Care Unit; PRISMA, Preferred Reporting Items for Systematic Reviews and Meta-Analyses; REML, Restricted Maximum Likelihood; TBI, Traumatic Brain Injury; US, Ultrasound / Ultrasonography.

## Data availability

The original contributions presented in the study are included in the article/Supplementary Material, further inquiries can be directed to the corresponding author.

The data that support the findings of this study are available from the corresponding author.

## Ethics statement

Ethical review and approval was not required for the study on human participants in accordance with the local legislation and institutional requirements.

## Funding

This review received no specific grant from any funding agency in the public, commercial, or not-for-profit sectors.

## Acknowledgement

This study was financed in part by the Coordenação de Aperfeiçoamento de Pessoal de Nível Superior – Brasil (CAPES) – Finance code 001.

## Conflicts of interest

The authors declare that there are no conflicts of interest regarding the publication of this article.
